# *Coreopsis tinctoria* Nutt. attenuates ultraviolet A photodamage by suppressing endoplasmic reticulum stress-induced apoptosis via Nrf2 crosstalk

**DOI:** 10.3389/fphar.2025.1686234

**Published:** 2025-12-12

**Authors:** Ling Liang, Xin Nie, Nan Zhao, Menggeng Li, Mingjie Li, Zhiwei Li, Man Wu, Xuanmin Wu, Cheuk-Lun Lee, Peng Shu, Jiangming Zhong

**Affiliations:** 1 HBN Research Institute and Biological Laboratory, Shenzhen Hujia Technology (Group) Co., Ltd., Shenzhen, Guangdong, China; 2 Department of Health Technology and Informatics, The Hong Kong Polytechnic University, Kowloon, Hong Kong SAR, China

**Keywords:** Coreopsis tinctoria, photodamage, endoplasmic reticulum (ER) stress, apoptosis, UVA, nuclear factor E2-related factor 2 (Nrf2)

## Abstract

**Background:**

Solar ultraviolet A (UVA) induces skin photodamage primarily by triggering endoplasmic reticulum (ER) stress, leading to misfolded protein accumulation and apoptosis. *Coreopsis tinctoria* Nutt. [Asteraceae] (CT), a medicinal chrysanthemum with antioxidant properties, has potential protective effects against UVA-induced skin injury. This study investigates the molecular mechanisms underlying CT’s photoprotective effects, emphasizing ER stress modulation and activation of antioxidant pathways.

**Methods:**

We characterized CT metabolites using UPLC-ESI-MS/MS, identifying 1,288 metabolites with flavonoids as predominant. The anti-UVA effects of CT in human keratinocytes (HaCaT) were assessed via bulk mRNA sequencing, Western blot analysis, immunofluorescence, flow cytometry, and siRNA-mediated knockdown of Nrf2. *In vivo*, UVA-irradiated murine models received topical CT treatment, with skin damage evaluated through immunohistochemistry and histopathology.

**Results:**

CT contains numerous flavonoids that contribute to its antioxidative capacity. In UVA-exposed HaCaT cells, CT significantly reduced apoptosis, inflammatory cytokine release, and reactive oxygen species (ROS) production. It downregulated ER stress markers (CHOP, pIRE1, ATF6), preserved ER morphology, and decreased downstream apoptotic signaling. Nrf2 knockdown experiments revealed that CT’s protective effects depend on Nrf2-mediated antioxidant responses. *In vivo*, topical CT application attenuated ER stress and skin injury induced by UVA exposure.

**Conclusion:**

CT alleviates UVA-induced skin photodamage by concurrently inhibiting all three branches of ER stress and activating Nrf2-driven antioxidant defenses, thereby modulating apoptosis. These findings position CT as a promising natural agent for dermocosmetic and therapeutic strategies against UVA-mediated skin injury.

## 1 Introduction

Skin photodamage refers to the harm caused by prolonged exposure to ultraviolet (UV) radiation from the sun or artificial sources. This damage can lead to various complications, including erythema (sunburn), inflammation, phototoxic dermatitis, photosensitive dermatitis, photoaging, and cancers (Tang et al., 2021). UV radiation is categorized into long-wave UVA (320–400 nm), medium-wave UVB (280–320 nm), and short-wave UVC (100–280 nm). The ozone layer absorbs most UVC, allowing only UVA (95%) and UVB (5%) to reach the Earth’s surface (D’Orazio et al., 2013). While UVB primarily affects the epidermis, UVA penetrates deeper to damage both the epidermis and dermal fibroblasts (Battie et al., 2014), posing a greater threat to skin health. UVA causes indirect DNA damage by generating reactive oxygen species (ROS) through cellular photosensitizers (Cadet et al., 2009). It also induces DNA lesions, including oxidized bases (8-oxo-dG), abasic sites, and single-strand breaks (Greinert et al., 2012). These lesions further activate signaling cascades such as nuclear factor-kappa B (NF-κB), leading to the production of proinflammatory cytokines (Montes de Oca et al., 2017). Additionally, UVA-generated ROS can trigger programmed cell death, such as apoptosis (Tang et al., 2021). Natural photoprotective compounds offer multifaceted benefits against UV-induced damage, not only by scavenging reactive oxygen species (ROS) but also through anti-inflammatory and signaling pathway modulation (Petruk et al., 2018). Supporting this, *Cornus mas* L. extract has been shown to attenuate UVA-induced oxidative stress, suppress melanogenesis, and reduce melanoma cell viability, indicating dual photoprotective and anti-pigmentation properties (Zagórska-Dziok et al., 2024). Similarly, a purified α-glucan from *Astragalus membranaceus* mitigates UVA-induced skin injury by decreasing ROS and restoring mitochondrial function, highlighting its potential as a multifunctional skincare agent (Li et al., 2020). Thus, there is a pressing need to explore intervention strategies to mitigate UVA-induced skin photodamage.

Endoplasmic reticulum (ER) stress occurs when protein folding is disrupted and the unfolded/misfolded proteins accumulate in the ER lumen. Disruptions in ER function, whether intrinsic or extrinsic, such as redox or calcium ion imbalances, can trigger ER stress and elicit the unfolded protein response (UPR) to maintain cellular protein homeostasis (Ma and Hendershot, 2004). However, prolonged and intense stimulation can cause excessive ER stress, resulting in cell apoptosis (Hetz et al., 2020). UV exposure is a known environmental factor that triggers ER stress and activation of the UPR by inducing photochemical generation of ROS (Farrukh et al., 2014). In the context of UV-induced skin photodamage and photoaging, the ER stress pathway is further activated in human dermal fibroblasts and keratinocytes, with the extent of activation positively correlated to the rate and intensity of UV exposure (Tai et al., 2024; Komori et al., 2012). Therefore, it is crucial to explore strategies for restoring ER homeostasis and alleviating ER stress following UVA exposure.

*Coreopsis tinctoria* (CT), an annual plant thriving in high-altitude mountainous regions, is primarily found in the coastal states of the Midwest, South, and Central Atlantic, as well as in the Xinjiang area of China (Shen et al., 2021). This plant is known for its diverse therapeutic properties, including antioxidant, anti-inflammatory, anti-diabetic, and neuroprotective effects (Guo et al., 2015). These properties are attributed to its rich composition of bioactive metabolites such as flavonoids (the most prevalent class), polyacetylenes, polysaccharides, phenylpropanoids, and volatile oils (Shen et al., 2021). Recent studies have highlighted the protective effects of okanin, a chalcone extract from CT, against UVB-induced skin damage in hairless mice (Sun et al., 2024). Okanin was shown to mitigate collagen degradation, oxidative stress, and inflammation. Additionally, marein, another chalconoid abundantly present in CT, inhibits NF-κB signaling activation in RAW264.7 *in vitro* (Li et al., 2022). Despite these findings, research on the efficacy of CT extracts in protecting against UVA-induced skin damage remains limited. In this study, we investigated the protective effects of CT against UVA-induced photodamage in human keratinocytes (HaCaT) via alleviating the ER stress and apoptosis. This conclusion is supported by metabolite profiling using UPLC-ESI-MS/MS and validated through a series of experiments ranging from *in vitro* assays in HaCaT cells—including bulk mRNA sequencing, Western blot, immunofluorescence, flow cytometry, and Nrf2 knockdown—to *in vivo* models assessed by immunohistochemistry.

## 2 Materials and methods

### 2.1 Preparation of bioactive metabolites from CT

The raw material, *Coreopsis tinctoria* Nutt. [Asteraceae] (CT) flower, was obtained from Guangxi Yifeng Pharmaceutical Co., Ltd (Guangxi, China). Fifteen kilograms of dried CT flowers were placed in an extraction tank with 300 L of purified water and extracted at 80 °C for 2 h. The first extraction solution was collected by filtration. An additional 225 L of purified water was added, and extraction was repeated for another 2 h to obtain the second extraction solution.

Both solutions were combined, centrifuged to remove insoluble impurities, and the supernatant was filtered through a 0.45 μm ceramic membrane (Plumem, Anhui, China). The permeate underwent ultrafiltration with a 5 kDa hollow fiber membrane, followed by nanofiltration with a 200 Da membrane to enrich total flavonoids and remove plant pigments. The retentate was concentrated under reduced pressure and dried, yielding 3.5 kg of CT extract. The extraction yield was 23.3% (w/w) on a dry weight basis (3.5 kg dry extract from 15 kg dry weight of plant material).

### 2.2 UPLC-ESI-MS/MS analyses of CT metabolites

For metabolomics analysis of the CT extract, the sample extracts were analyzed using an UPLC-ESI-MS/MS system (ExionLC™ AD, SCIEX) coupled with a QTRAP mass spectrometer. Chromatographic separation was performed on an Agilent SB-C18 column (1.8 µm, 2.1 × 100 mm) maintained at 40 °C. The mobile phase consisted of (A) 0.1% formic acid in water and (B) 0.1% formic acid in acetonitrile, delivered at a flow rate of 0.35 mL/min with the following gradient program: 0–1 min, 5% B (v/v); 1–9 min, 5%–95% B; 9–10 min, hold for 1 min, 95% B; 10–11 min, decreased to 5% B; 11–25 min, 5% B (equilibration). The injection volume was 2 μL.The ESI source was operated at 500 °C with ion spray voltages of 5500 V (positive mode) and −4500 V (negative mode). Gas parameters were set as follows: ion source gas I (50 psi), gas II (60 psi), and curtain gas (25 psi). The collision-activated dissociation was set to high. MRM experiments were performed with nitrogen as collision gas (medium setting), with optimized declustering potential (DP) and collision energy (CE) for individual transitions. Specific MRM transitions were monitored according to metabolite elution profiles.

For the quantitative analysis of representative metabolites in CT extract, a UPLC-MS/MS system was used, consisting of a Waters ACQUITY UPLC H-Class (Waters, America) and a Sciex QTRAP 4500 (Sciex, America). A C18 column (2.1 × 150 mm, 2.5 μm) was used with a mobile phase composed of solvent A (methanol) and solvent B (water) under the following gradient elution program: 0–2 min, 10% A; 2–6 min, 10%–80% A; 6–8 min, 80%–10% A; 8–10 min, 10% A, at a flow rate of 0.3 mL/min and column temperature of 30 °C. Mass spectrometric detection was performed in negative electrospray ionization (ESI-) mode with the ion spray voltage set at −4500 V, GS1 at 50.0, GS2 at 55.0, curtain gas at 40.0, source temperature at 550 °C, and collision activated dissociation (CAD) at medium pressure. The selected reaction monitoring (SRM) transitions were as follows: chlorogenic acid (353 > 190.9), flavanomarein (449 > 286.8), taxifolin (303 > 285), okanin (287 > 134.9), marein (449 > 286.9), and isookanin (287 > 134.9). The selected reaction monitoring (SRM) transitions were as follows: gallic acid (169 > 124.8), caffeic acid (179 > 135), vanillic acid (167 > 107.9), myricetin (317 > 150.9), quercetin (301 > 150.8), butein (271 > 134.9), luteolin (285 > 132.9), kaempferol (285 > 159), apigenin (269 > 116.8), chlorogenic acid (353 > 190.9), flavanomarein (449 > 286.8), taxifolin (303 > 285), okanin (287 > 134.9), marein (449 > 286.9), and isookanin (287 > 134.9). The metabolites in the samples were analyzed both qualitatively and quantitatively by using Analyst software 1.7.3 (SCIEX, America) and SCIEX OS.

### 2.3 Cell culture and UVA radiation

Human keratinocyte cell line HaCaT was sourced from EK-Bioscience, China. The cells were seeded in a 6-well plate and cultured in Dulbecco’s Modified Eagle’s Medium (DMEM, Gibco) supplemented with 10% fetal bovine serum (FBS, Gibco) and 1% penicillin–streptomycin (P/S, Gibco) in a humidified atmosphere containing 5% CO_2_ at 37 °C. Prior to UVA irradiation, the cells were washed and then overlaid with 1 mL of phosphate-buffered saline (PBS). The HaCaT cells were then exposed to UVA radiation (365 nm) for 300 s at a dose of 5 J/cm^2^ using a UVA crosslinker (UCL-3500L, Luyor), followed by treatment with specified concentrations (50 μg/mL, 100 μg/mL) of CT extracts (Chen et al., 2006). Control groups underwent the same medium change schedule without UVA irradiation.

### 2.4 Cell viability assay

HaCaT cells were seeded into 96-well plates and subjected to the indicated treatments. Cell viability was performed as reported previously using the Cell Counting Kit-8 (CCK8) (Beyotime, C0043) (Zhong et al., 2025). Briefly, 1 mL of 2-(2-methoxy-4-nitrophenyl)-3-(4-nitrophenyl)-5-(2,4-disulfophenyl)-2H-tetrazolium monosodium salt was added to each well, and the plates were incubated at 37 °C for 2 h. Absorbance was measured at 450 nm with a reference wavelength of 650 nm, and the results were normalized to those of the control groups.

### 2.5 Cell apoptosis assay

The apoptosis ratio was analyzed as reported previously using the Annexin V-FITC Apoptosis Detection Kit (Beyotime, C1062L) (Zhong et al., 2025). Cells were harvested and resuspended in a buffer containing Annexin V-FITC and propidium iodide (PI) according to the manufacturer’s instructions. Flow cytometry analysis was performed using a flow cytometer (Beckman, CytoFlex, United States of America), and data were analyzed with CytExpert software (Beckman, United States of America).

### 2.6 Total RNA extraction and quantitative PCR (qPCR) analysis

Total RNA was extracted from the cells using the TransZol Up Plus RNA Kit (Transgen). The extracted RNA was then reverse-transcribed into cDNA using the 5X Evo M-MLV RT Reaction Mix (Agbio, AG11728). The resulting cDNA products were used as quantitative PCR (qPCR) analysis templates. The qPCR reactions were performed using the SYBR Mix (Yeasen, 11202 ES). The sequences of the primer pairs used in the qPCR analysis are provided in Table 1.

**TABLE 1:** Primer sequences used for qPCR analysis.

Gene	Forward primer (5'→3′)	Reverse primer (5'→3′)
β-Actin	CAT​GTA​CGT​TGC​TAT​CCA​GGC	CTC​CTT​AAT​GTC​ACG​CAC​GAT
IL-6	ACT​CAC​CTC​TTC​AGA​ACG​AAT​TG	CCA​TCT​TTG​GAA​GGT​TCA​GGT​TG
IL-1β	ATG​ATG​GCT​TAT​TAC​AGT​GGC​AA	GTC​GGA​GAT​TCG​TAG​CTG​GA
TNF-α	CCT​CTC​TCT​AAT​CAG​CCC​TCT​G	GAG​GAC​CTG​GGA​GTA​GAT​GAG

### 2.7 Western blot analysis

Total cellular proteins were extracted using RIPA buffer (Beyotime, P0025) with the application of proteasome inhibitor (MG-132, MCE, United States). Protein extracts were then denatured by 5× SDS loading buffer (Beyotime, P0015L), separated by SDS-PAGE, and transferred to PVDF membrane (Millipore, IPVH00010). The membranes were blocked with 5% non-fat milk and incubated with the following primary antibodies: IRE1 (phospho S724) (Abcam, ab124945); CHOP (Proteintech,15204-1-AP); NRF2 (Proteintech, 16396-1-AP); ATF6 (Proteintech, 24169-1-AP); PERK (phospho Thr982) (Proteintech, 82534-1-RR); eIF2α(phospho Ser51) (Proteintech, 68023-1-Ig); GADD34 (Proteintech, 10449-1-AP); Caspase9 (Proteintech, 10380-1-AP); Cleaved caspase3 (Proteintech, 25128-1-AP); JNK (Proteintech, 51153-1-AP); JNK (phospho Tyr185) (Proteintech, 80024-1-RR); Bax (Proteintech, 50599-2-Ig); BCL2 (Proteintech, 60178-1-Ig); HO-1 (Proteintech, 10701-1-AP); β-Actin (Proteintech, 20536-1-AP); Horseradish peroxidase (HRP)-conjugated goat anti-rabbit and anti-mouse secondary antibodies were applied (Jackson, 111-035-003 and 115-035-003). The bands were visualized by super-sensitive ECL luminescence reagent (Meilunbio, MA0186-2), and the images were captured using an eBlot system (Genscript). ImageJ (NIH, United States) was applied to analyze the gray density to quantify protein expression.

### 2.8 ER-tracker

ER-Tracker Green kit (Beyotime, C1042S) was used to visualize the endoplasmic reticulum. Cells were cultured in a 24-well plate with glass coverslips. After treatment, diluted ER tracker was incubated with cells for 30 min at 37 °C. The cells were then washed for two times and nuclei were counter-stained with DAPI (Roche, United States). The glass coverslips were removed and cell images were taken on a fluorescent microscope (DMi8, Leica, Germany).

### 2.9 Immunofluorescence staining

Cells were fixed in 4% (v/v) paraformaldehyde for 10 min for immunofluorescence staining. The cells were permeabilized with 0.5% (v/v) Triton X-100 for 10 min and subsequently blocked with 5% (v/v) BSA for 1 h at room temperature. Following three washes with PBS, the cells were incubated overnight at 4 °C with the primary antibody CHOP (Proteintech,15204-1-AP). Afterward, they were incubated for 1 h at room temperature with an Alexa Fluor 488-conjugated goat anti-rabbit secondary antibody (Life Technologies). Nuclei were counterstained with DAPI (Beyotime, China). Cell images were captured using a fluorescent microscope (DMi8, Leica, Germany).

### 2.10 Mitochondrial membrane potential assay

Cells were collected and incubated using an Enhanced Mitochondrial Membrane Potential Assay Kit with the cyanine dye JC-1 (5,5′,6,6′-tetrachloro-1,1′,3,3′-tetraethylbenzimi- dazolylcarbocyanine iodide, Beyotime, C2003S) according to the manufacturer’s protocol. Briefly, HaCaT cells (5 × 10^5^) were harvested and washed with PBS. The cells were then incubated with the JC-1 probe at 37 °C in the dark for 30 min. Following incubation, the cells were washed three times with JC-1 staining buffer.

The mitochondrial membrane potential was assessed using a microplate reader (Tecan Infinite E PLEX, Switzerland). For detection of the JC-1 monomer, the excitation wavelength was set to 490 nm and the emission wavelength to 530 nm. For detection of JC-1 polymers, the excitation wavelength was set to 525 nm and the emission wavelength to 590 nm.

### 2.11 Bulk mRNA sequencing

HaCaT cells were treated as described earlier. Total RNA was extracted using TRIzol™ Reagent (Gibco, 15596026CN) according to the manufacturer’s protocol. VAHTS mRNA capture beads attached to Oligo (dT) were used to enrich and purify eukaryotic mRNA. RNA was fragmented to 250-450 bp and converted into a cDNA library. The quality of the library was evaluated using Qubit 4.0, LabChip, or FA and ABI QuantStudio 12K quantitative QPCR. RNA-seq was performed using the Illumina Novaseq™ 6000 platform. Gene abundance was quantified using FPKM (fragments per kilobase of exon model per million mapped reads). For differential and significant gene expression analysis, threshold for significantly upregulated changes was set at a fold change (FC) > 2 with a statistically significant p-value <0.05, while significantly downregulated changes were defined as FC < −2 with a p-value <0.05.

### 2.12 Transient transfection with Nrf2 siRNA

NRF2 knockdown in HaCaT cells was performed using Lipofectamine™ 3,000 Reagent (Invitrogen, L3000015) with the following siRNA sequences: sense 5'→3′GGU​UGA​GAC​UAC​CAU​GGU​UTT, antisense 5'→3′AAC​CAU​GGU​AGU​CUC​AAC​CAG. Briefly, cells were seeded to reach 70%–90% confluency at transfection. Lipofectamine™ 3,000 reagent was diluted in Opti-MEM Medium and mixed. The siRNA was diluted in Opti-MEM Medium (Gibco, 31,985,070), then P3000™ Reagent was added and mixed. The diluted siRNA was combined with diluted Lipofectamine™ 3,000 Reagent and incubated for 20 min at room temperature. The siRNA-lipid complexes were added to cells for 24 h, after which the medium was replaced with DMEM (high glucose) or other treatments.

### 2.13 Animal

BALB/c mice (male, 18–22 g, 6 weeks) were obtained from BesTest Bio-Tech Co,.Ltd (China) and maintained under standard laboratory conditions in compliance with the Guidelines for the Care and Use of Laboratory Animals (GB/T 16886.2–2011) (Animal Ethics Approval code: K2025-02-171-046; the animal use permit number: SCXK (Guangdong) 2020-0051). The animals were housed in a conventional animal facility with a controlled 12-h light/dark cycle. Room temperature was maintained at 18 °C–26 °C, and relative humidity was regulated between 40% and 70%, with daily monitoring to ensure stability.

### 2.14 UVA mice model establishment and CT treatment

BALB/c mice were randomly divided into three experimental groups (n = 6 per group): Control Group, UVA-Treated Group, and UVA + CT-Treated Group. After a 5-day acclimatization period, the dorsal hair of the mice was shaved to expose a 3 × 3 cm^2^ treatment area. The UVA irradiation time (T) was calculated to deliver a cumulative dose of 5 J/cm^2^ using a UVA irradiation device (365 nm) (Beijing Aerospace Hong Da Optoelectronics Technology Co., Ltd., Model AHD-500W-T/2/500W). The calculation was performed using the formula: T (s) = Total irradiation dose (J/cm^2^) ÷ Irradiation area (cm^2^) ÷ Irradiation intensity (μW/cm^2^) × 1,000. Mice in the experimental groups received daily UVA exposure for 7 consecutive days to establish the photodamage model. Drug administration was performed 30 min post-UVA irradiation from day 8 to day 14. The UVA-Treated Group received no additional treatment, while the UVA + CT-Treated Group was topically administered 0.3 g of a 1% CT-containing formulation once daily. The composition of the formulation containing 1% CT is shown in Supplementary Figure S1B.

### 2.15 Hematoxylin and eosin (H&E) staining and Masson staining

After 14 days of irradiation, the central area of the skin from mice was harvested and fixed in 4% paraformaldehyde solution (Beyotime, P0099). Subsequently, the tissue was dehydrated using an automated tissue processor (LEICA, ASP300S), embedded with a tissue embedding machine (Taiva, TB-718E), and sectioned into 5 µm using a semi-automatic rotary microtome (LEICA, Histo Core MULTICUT). The sections were then mounted and dried using a thermostatic slide warmer/dryer (Taiva, TK-218II). Hematoxylin and eosin (H&E) staining as well as Masson’s trichrome staining were performed using a staining machine (Taiva, TR-180I).

### 2.16 Immunohistochemical staining

After dewaxing with xylene (Macklin, X821391), the slides were subjected to ethanol (XIHUA, 1000874) gradient rehydration, followed by heat-induced antigen epitope retrieval using Tris-EDTA antigen retrieval buffer (Proteintech, PR30002). After incubation with an endogenous peroxidase blocker (ZSGB-BIO, PV-6000), the slides were blocked with 3% BSA for 1 h. Primary antibodies against NRF2 (Proteintech, 16396-1-AP), Caspase12 (Proteintech, 55238-1-AP), and CHOP (Proteintech, 15204-1-AP) were incubated overnight at 4 °C at a dilution of 1:200 each. The secondary antibody, HRP-conjugated goat anti-mouse/rabbit IgG polymer (ZSGB-BIO, PV-6000), was incubated for 20 min at 37 °C. DAB chromogenic reagent kit (ZSGB-BIO, ZLI-9017) was used for color development, and the cell nuclei were counterstained with hematoxylin (Beyotime, C0105). Images were captured using a microscope (DMi8, Leica, Germany).

### 2.17 Statistical analysis

Statistical analysis was performed using GraphPad Prism (version 9.0; GraphPad Software Inc.). The significance of differences in variables between groups was assessed using one-way analysis of variance (one-way ANOVA). The Tukey method was used to adjust multiple comparisons. The data are expressed as the means ± standard deviations, all statistical tests were two-sided, and p < 0.05 was considered statistically significant; *p < 0.05, **p < 0.01, ***p < 0.001.

### 2.18 Free radical scavenging activity

The radical scavenging activity of CT extract was determined using the ABTS assay, which was performed according to a previously reported method (Re et al., 1999). Briefly, 7.4 mM ABTS and 2.4 mM potassium persulfate solutions were mixed in a 1:1 ratio and incubated overnight at room temperature to generate ABTS radicals. Before use, the ABTS solution was diluted with distilled water until its absorbance (OD) at 734 nm reached 0.70 ± 0.02. Different concentrations of CT extract (0–400 μg/mL) were then mixed with the ABTS solution, transferred to a 96-well plate, and incubated for 6 min at room temperature. Absorbance was measured at 734 nm. The ABTS scavenging effect was calculated using the following formula:

$$\text{ABTS scavenging effect }\left(\%\right)=\left[\left(A_{0}-A_{1}\right)/A_{0}\right]\times 100$$

where A_0_ is the absorbance of ABTS, and A_1_ is the absorbance of samples.

### 2.19 Cellular ROS production detection

Cellular ROS production was measured using the Reactive Oxygen Species Assay Kit (Beyotime, S0033M). Briefly, DCFH-DA was diluted with PBS at a 1:1,000 ratio to obtain a final concentration of 10 µM. After removal of the cell culture medium, an appropriate volume of the diluted DCFH-DA was added to cover the cells, followed by incubation at 37 °C for 20 min in a cell culture incubator. Subsequently, the cells were washed three times with PBS. The ROS level was measured using a microplate reader (Tecan Infinite E PLEX, Switzerland) with excitation and emission wavelengths set at 490 nm and 525 nm. Quercetin (ShanghaiyuanyeBio-Technology, A10009) was used as a positive control at a concentration of 20 μM according to a previously reported (Zhu et al., 2017).

## 3 Results

### 3.1 Characterization of the CT extract

**FIGURE 1:**
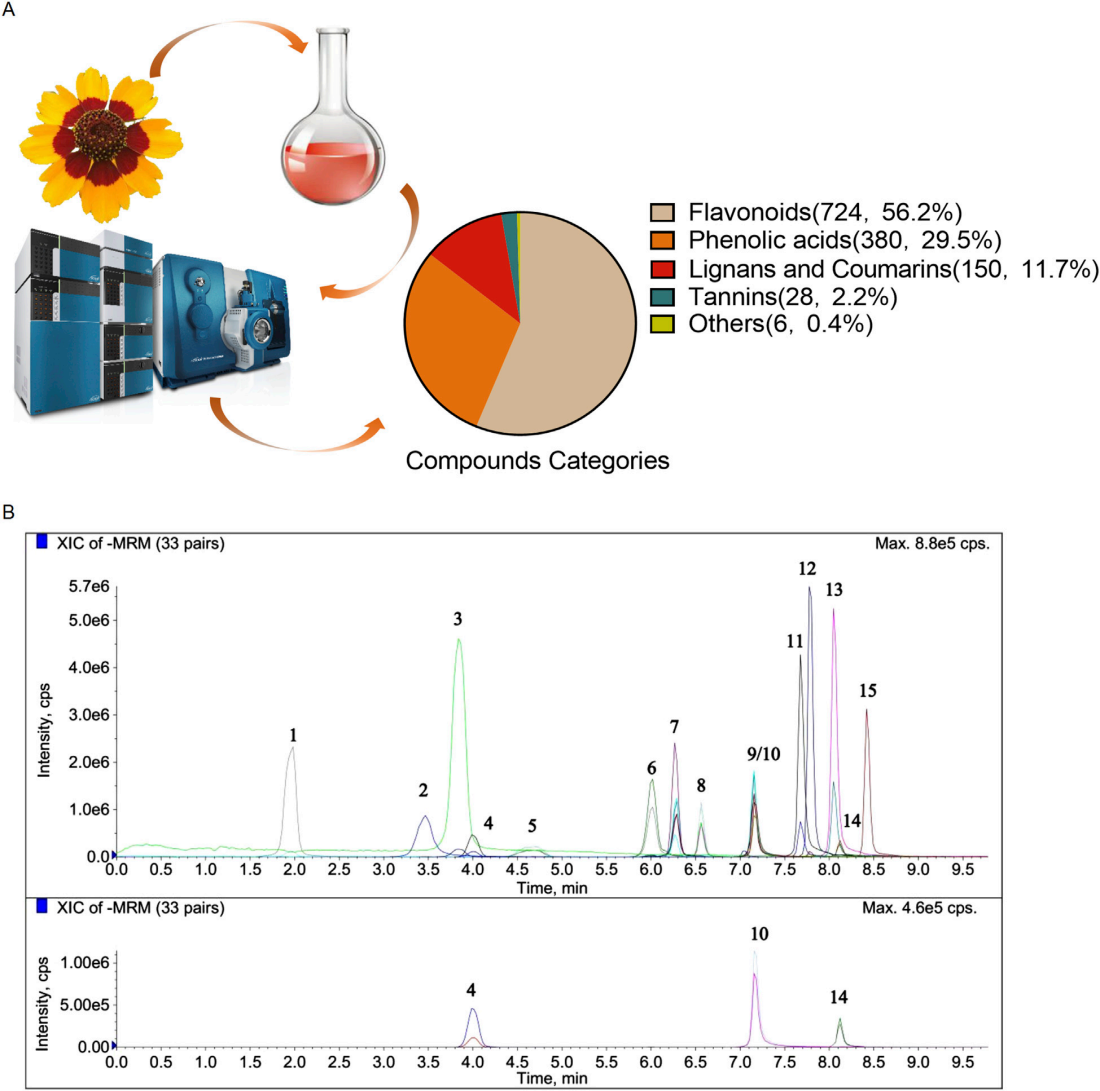
Characterization of the CT extract. **(A)** Schematic representation of the experimental design to characterize CT metabolites by UPLC-ESI-MS/MS. **(B)** Chromatogram analysis of CT metabolites using the UPLC-MS/MS method.

By applying a UPLC-ESI-MS/MS system for total metabolomics analysis in CT exract, a total of 1,288 metabolites were identified from both positive and negative ionization modes (Supplementary Table S1). Especially, flavonoids were the most predominent metabolites with 724 were characterized. Other metabolites included 380 phenolic acid, 150 lignans and coumarins, 28 tannins and 6 others (Figure 1A). Compared with the metabolites previously identified in other CT samples, our study substantially enriches the understanding of the types and constituents of metabolites in CT (Wang et al., 2022). As shown in Figure 1B and Table 2, we further characterized 15 representative metabolites in comparison with pure standards. Despite differences in the botanical origins of CT and the extraction methods used, our analysis identified the same compounds—including chlorogenic acid, caffeic acid, vanillic acid, taxifolin, isookanin, marein, myricetin, quercetin, butein, luteolin, kaempferol, and apigenin—as those reported in CT extracts by other researchers (Wang et al., 2022) (Ma et al., 2022; Jiang et al., 2022).

**TABLE 2:** Name of the metabolites with their molecular formulas, calculated molecular weight, mass to charge (*m*/*z*) ratios, retention times, peak area and the content in the extract of the representative metabolites.

Number	Name	Formula	Calc. MW	m/z	RT (min)	Area	Content in the extract (mg/g)
1	Gallic acid	C7H6O5	170.12	169	2.02	201,000	0.0108
2	Chlorogenic acid	C16H18O9	354.31	353	3.61	373,000	3.620
3	Caffeic acid	C9H8O4	180.16	179	3.99	618,000	0.156
4	Vanillic acid	C8H8O4	168.149	167	4.13	68,500	0.0225
5	Flavanomarein	C21H22O11	450.39	449	4.91	3,030,000	69.600
6	Taxifolin	C15H12O7	304.25	303	6.11	857,000	0.936
7	Isookanin	C15H12O6	288.3	287	6.34	1,560,000	20.200
8	Marein	C21H22O11	450.39	449	6.60	18,200,000	40.100
9	Okanin	C15H12O6	288.25	287	7.18	32,800,000	4.100
10	Myricetin	C15H10O8	318.24	317	7.19	67,200	0.126
11	Quercetin	C15H10O7	302.24	301	7.69	743,000	0.386
12	Butein	C15H12O5	272.25	271	7.79	470,000	0.175
13	Luteolin	C15H10O6	286.24	285	8.06	411,000	0.180
14	Kaempferol	C15H10O6	286.24	285	8.13	35,300	0.0069
15	Apigenin	C15H10O5	270.24	269	8.43	196,000	0.00667

### 3.2 CT protects HaCaT cells against UVA-induced photodamage

**FIGURE 2:**
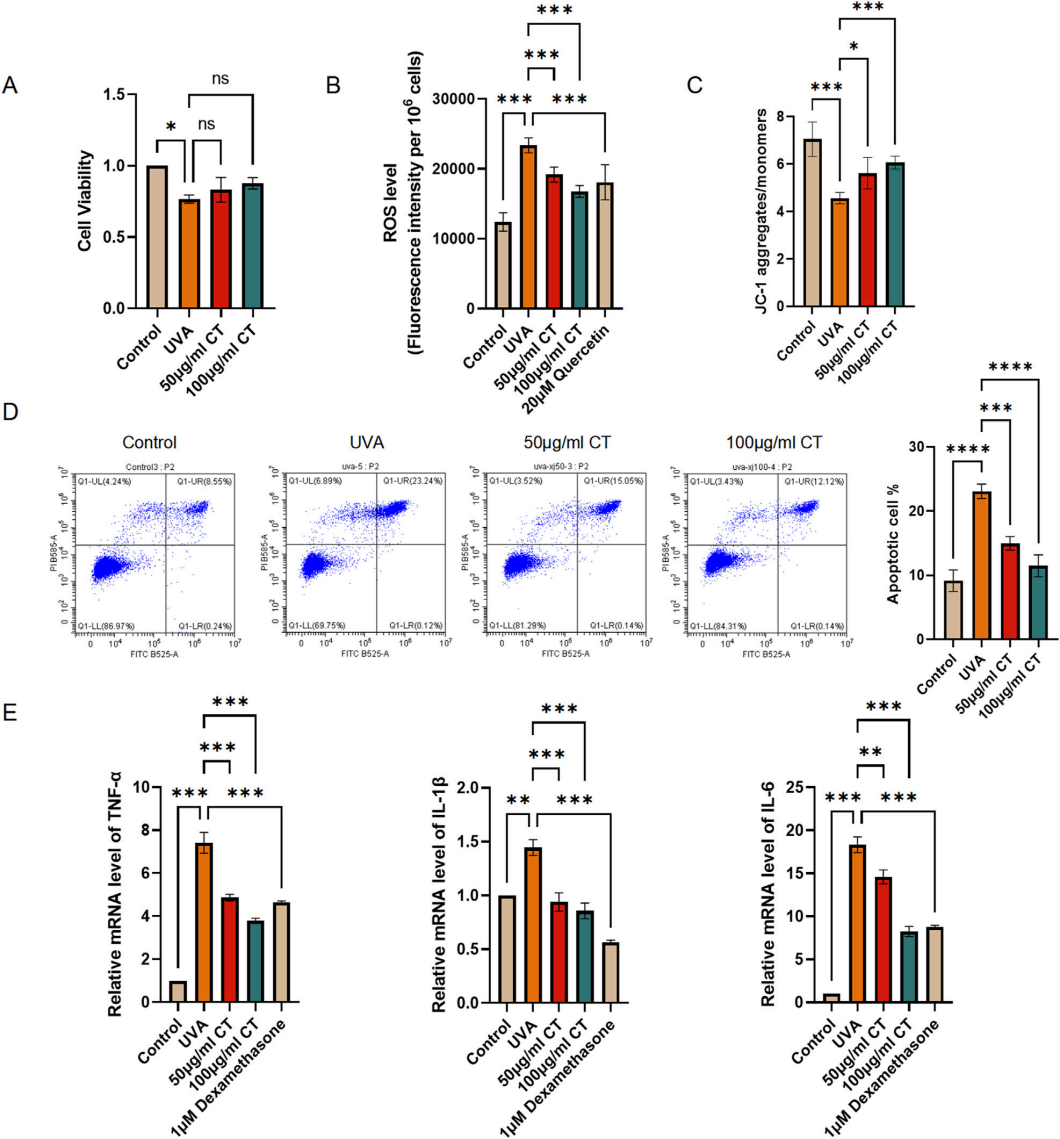
CT extract protects HaCaT cells against UVA-induced photodamage. HaCaT cells were subjected to UVA (5 J/cm^2^) radiation and then treated with the indicated concentrations of extraction from CT. **(A)** CCK-8 assay was performed to determine cell viability in different treatments. **(B)** ROS level was determined in different treatments. **(C)** JC-1 assessing mitochondrial membrane potential was determined in different treatments. **(D)** Flow cytometry analysis was performed to determine cell apoptosis. Quantification of the percentage of apoptotic cells in different treatments. **(E)** RT-qPCR assays were performed to determine the mRNA levels of IL-1β, IL-6, and TNF-α. N = 4. *p ≤ 0.05; **p ≤ 0.01, ***p ≤ 0.001.

To assess the cell viability of HaCaT cells following UVA-induced photodamage with/without CT treatment, we performed a CCK-8 assay. HaCaT cells were exposed to UVA radiation (5 J/cm^2^) and treated with various concentrations of CT (50 and 100 μg/mL). As illustrated in Figure 2A, treatment with 50 μg/mL and 100 μg/mL of CT was chosen to conduct the following experiments as the cell viability after UVA exposure was above 80% and had no significant difference with the control group.

UVA radiation is known to generate ROS and decrease mitochondrial membrane potential, leading to oxidative stress, cellular damage, and eventually apoptosis (Jaszewska et al., 2013). To further investigate the effects of CT, we measured ROS production, alterations in mitochondrial membrane potential, and cellular apoptosis in response to UVA exposure. Our findings indicated that both 50 and 100 μg/mL of CT effectively reduced intracellular ROS levels in UVA-irradiated HaCaT cells, with efficacy comparable to the positive control quercetin (Figure 2B). Meanwhile, the *in vitro* biochemical assay, ABTS, demonstrated that CT itself possesses potent inherent antioxidant activity (Supplementary Figure S1A). Additionally, mitochondrial depolarization, indicated by a decrease in the aggregate/monomer ratio from the JC-1 assay, was reversed by treatment with 50 and 100 μg/mL of CT, restoring mitochondrial membrane potential (Figure 2C). Furthermore, apoptosis assays demonstrated that both 50 and 100 μg/mL of CT mitigated UVA-induced apoptosis in HaCaT cells (Figure 2D).

Previous studies have indicated that UV-induced photodamage is linked to the release of inflammatory molecules such as IL-1β, IL-6, and TNF-α from epidermal keratinocytes (Ansary et al., 2021). To explore this further, we examined the impact of CT and positive control dexamethasone (1 μM) on inflammatory cytokine levels (Li et al., 2016). As shown in Figure 2E, the mRNA levels of IL-1β, IL-6, and TNF-α were significantly reduced in HaCaT cells treated with 100 μg/mL of CT compared to UVA-irradiated HaCaT cells without CT treatment.

Taken together, our findings suggest that CT protects HaCaT cells from UVA-induced photodamage via mechanisms involving the modulation of ROS levels, mitochondrial membrane potential, apoptosis, and the release of inflammatory cytokines.

### 3.3 mRNA sequencing for the effects of CT on UVA-irradiated response in HaCaT

**FIGURE 3:**
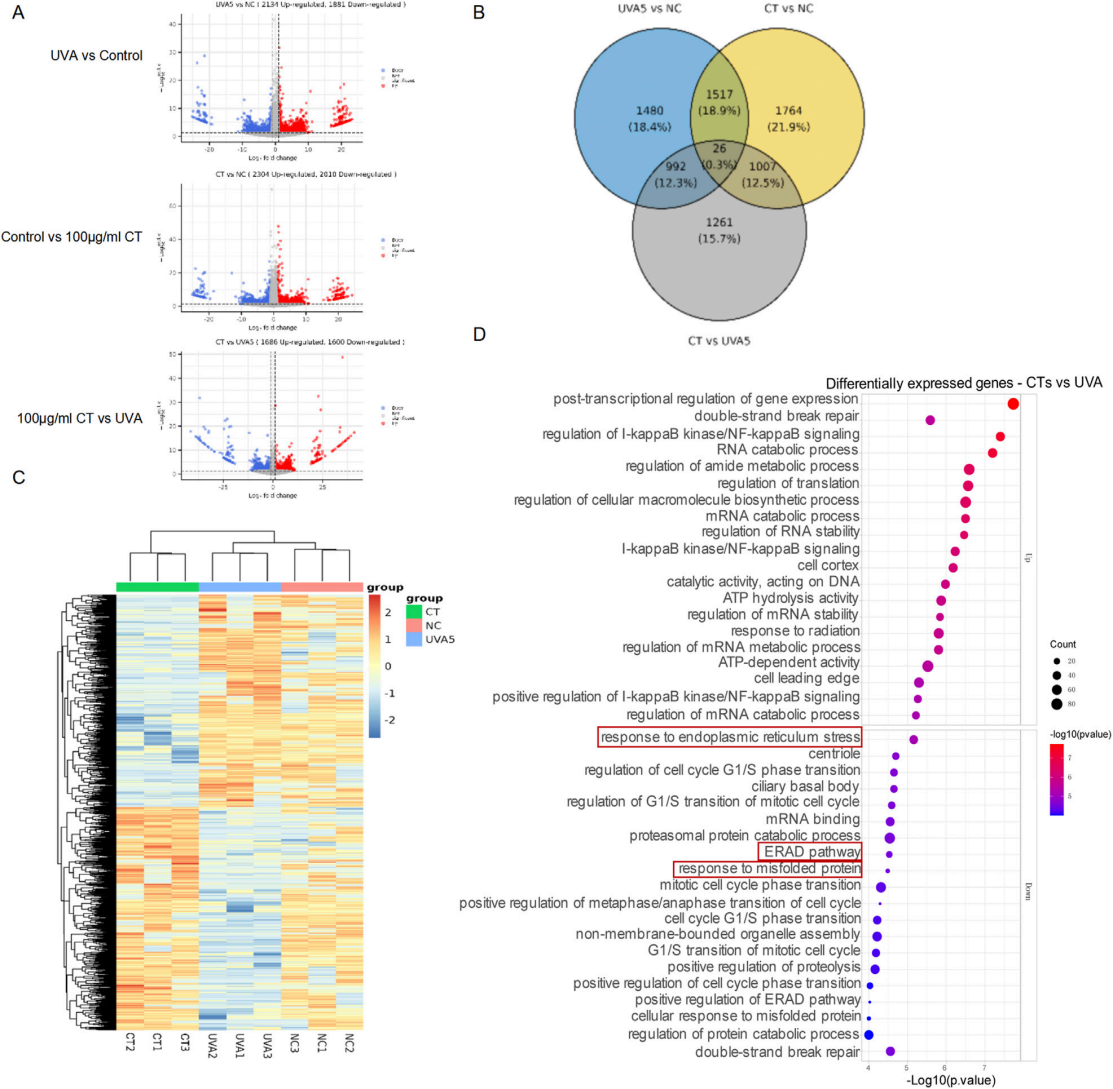
mRNA sequencing for the effects of CT on UVA-irradiated response in HaCaT. **(A)** Volcano plots of gene expression in transcriptome profiling of UVA-irradiated HaCaT cells (UVA), CT-treated UVA-irradiated HaCaT cells (CT), and control HaCaT cells (NC). Significantly upregulated and downregulated genes are highlighted in red and blue, respectively. **(B)** The Venn diagram shows the overlap genes of the UVA-irradiated HaCaT cells, CT-treated UVA-irradiated HaCaT cells, and control HaCaT cells. **(C)** Heatmap shows hierarchical clusters based on UVA-irradiated HaCaT cells, CT-treated UVA-irradiated HaCaT cells, and control HaCaT cells. **(D)** GO enrichment analysis of DEGs between UVA-irradiated HaCaT cells and CT-treated UVA-irradiated HaCaT cells.

We conducted mRNA sequencing on the following groups: UVA-irradiated HaCaT cells, 100 μg/mL of CT -treated UVA-irradiated HaCaT cells, and control HaCaT cells. As shown in Figures 3A,B, UVA exposure induced 4,015 differentially expressed genes (DEGs) compared to the control group. Treatment with CT with UVA exposure resulted in 4,313 DEGs relative to the control group and 3,286 DEGs compared to the UVA-exposed group. Hierarchical clustering demonstrated that the UVA-irradiated HaCaT cells, CT-treated UVA-irradiated HaCaT cells, and control HaCaT cells formed distinct clusters, separate from their respective controls (Figure 3C). Furthermore, the DEGs between CT and UVA underwent Gene Ontology (GO) enrichment analysis, revealing that the ‘response to ER stress’, ‘cellular response to misfolded proteins’ and ‘ERAD (ER-associated degradation) pathway’ were significantly enriched among the DEGs between UVA-irradiated HaCaT cells and CT-treated UVA-irradiated HaCaT cells (Figure 3D).

### 3.4 CT decreases UVA-Induced ER stress in HaCaT cells

Previous studies have indicated that UV-induced cell apoptosis in the skin is triggered by excessive ER stress, which mediates the unfolded protein response (Tai et al., 2024). Our bulk RNA sequencing analysis further supported this mechanism, revealing that CT’s protective effects against UVA damage were partially associated with response to ER stress. Based on these findings, we specifically examined CT’s capacity to ameliorate UVA-induced ER stress.

**FIGURE 4:**
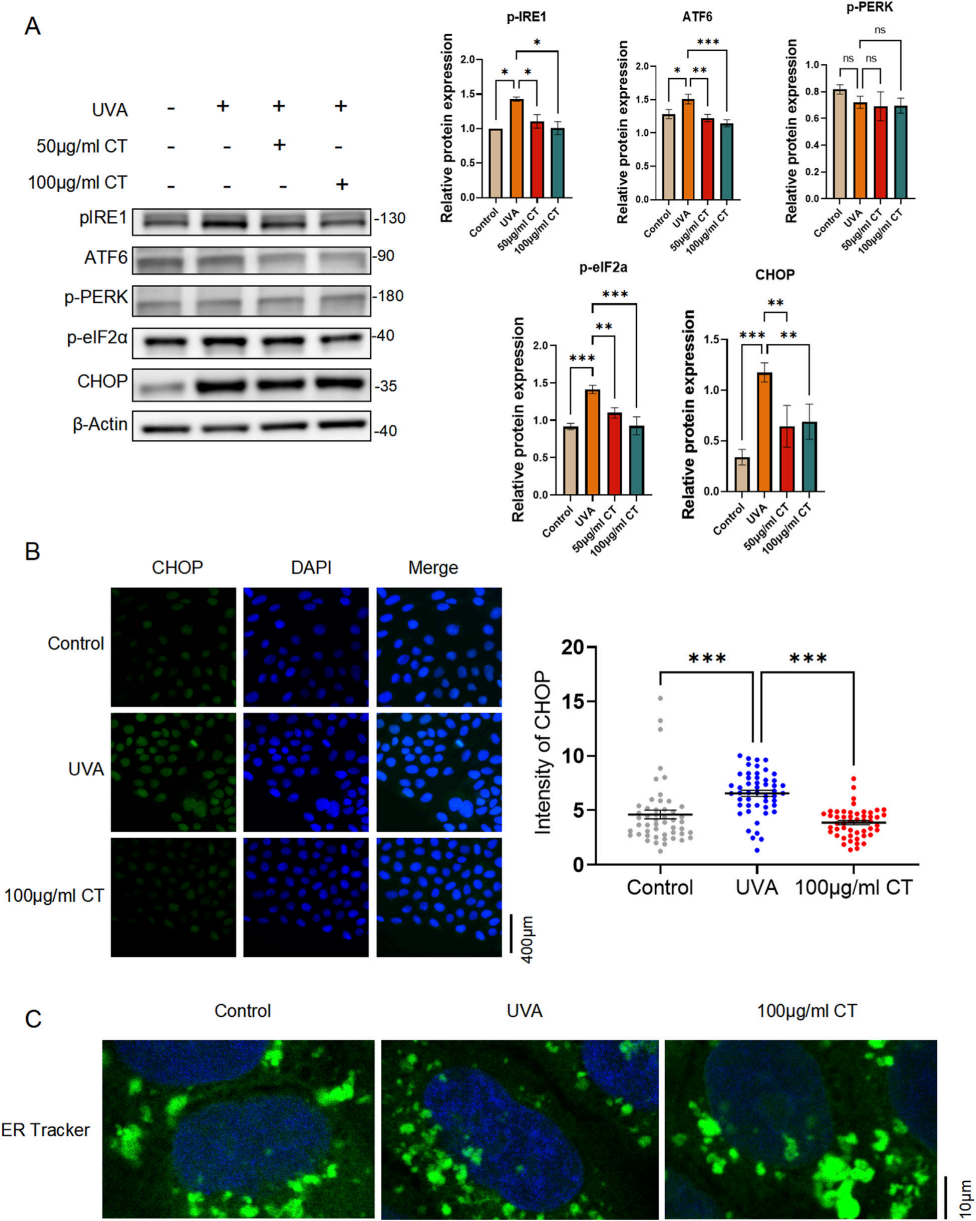
Extraction from CT decreases UVA-induced ER stress in HaCaT cells. HaCaT cells were subjected to UVA (5 J/cm^2^) radiation and then treated with the indicated concentrations of extraction from CT. **(A)** Western blot analysis was performed to determine the protein levels of phosphorylated IRE1 (pIRE1), ATF6, phosphorylated PERK (p-PERK), phosphorylated eIF2a (p-eIF2a) and CHOP in different treatments. **(B)** Immunofluorescence assay was performed to detect CHOP expression. **(C)** Representative images showing the ER morphology in control, UVA-treated and CT-treated cells with UVA exposure. Cells were stained with ER tracker in green and counterstained with DAPI for nuclear visualization. N = 4. *p ≤ 0.05; **p ≤ 0.01, ***p ≤ 0.001.

**FIGURE 5:**
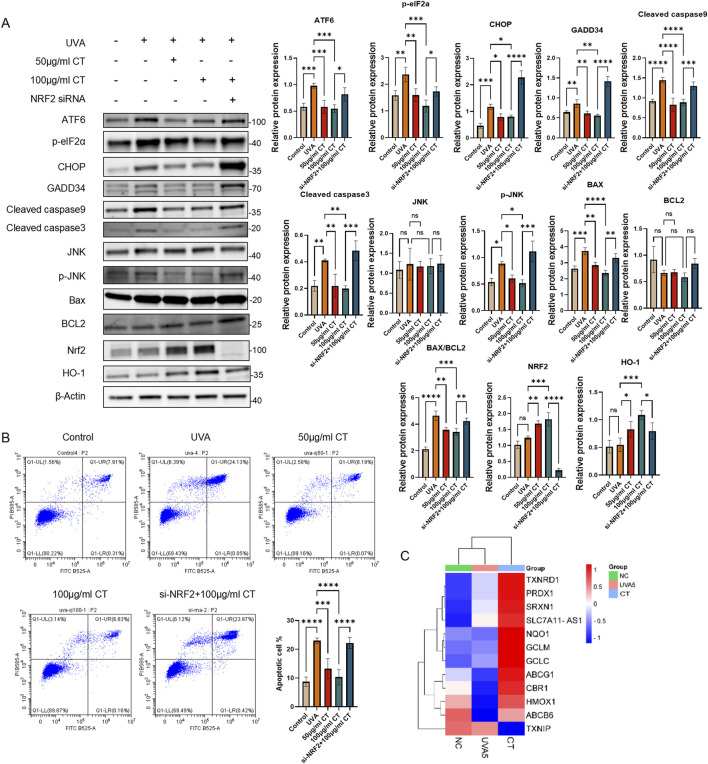
CT alleviates UVA-induced ER stress and apoptosis via Nrf2 in HaCaT cells. **(A)** HaCaT cells were subjected to UVA radiation and treated with 50 or 100 μg/mL CT and/or siNrf2. Western blot assays were performed to detect the levels of ATF6, phosphorylated elF2a (p-elF2a), CHOP, GADD34, Cleaved caspase9, Cleaved caspase3, JNK, phosphorylated JNK (p-JNK), Bax, BCL2, cytoplasmic and nuclear NRF2 and HO-1. **(B)** Flow cytometry analysis was performed to determine cell apoptosis. Quantification of the percentage of apoptotic cells in different treatments. **(C)** Heatmap generated the data from RNAseq. Genes are all related to NRF2 downstream anti-oxidant or detoxification target genes. The figure was generated by SRplot (Tang et al., 2023). N = 4. *p ≤ 0.05; **p ≤ 0.01, ***p ≤ 0.001, ****p ≤ 0.0001.

Cellular markers of ER stress induction, including phosphorylated IRE1 (pIRE1), ATF6, phosphorylated PERK (p-PERK), phosphorylated eIF2a (p-eIF2a) and CHOP, were selected to determine the extent of ER stress. As shown in Figures 4A, 5 μg/mL and 100 μg/mL of CT both significantly reduced UVA-induced elevation of ER stress by downregulating the protein expression of pIRE1, ATF6, p-eIF2a and CHOP elicited by UVA irradiation.

Since the major component of the ER stress-mediated apoptosis pathway is CHOP (Li et al., 2014), we then conducted an immunofluorescence assay of CHOP. As indicated in Figure 4B, there was an overall downregulation of CHOP expression intensity after 100 μg/mL of CT treatment upon the UVA exposure.

Changes in ER morphology correlate with the occurrence of ER stress (Guo et al., 2021). Using an ER tracker, we observed the emergence of small fragmented ring-shaped ER whorls after UVA treatment, indicating localized ER stress. This phenomenon was abolished after the addition of 100 μg/mL CT, and the ER morphology returned to a continuous envelope with a network of interconnected tubules and sheets (Figure 4C).

### 3.5 Nrf2 knockdown revealed that CT alleviated UVA-Induced ER stress and apoptosis through antioxidant Nrf2

Given that UVA exposure can lead to prolonged ER stress and subsequent pro-apoptotic signaling, we investigated the potential of CT to counteract ER stress-related apoptosis. The antioxidative Nrf2/HO-1 pathway has been reported to play a critical role in regulating ER stress (Zhang et al., 2022; Yu et al., 2022). To explore this further, we hypothesized the CT effects in modulating the ER stress was Nrf2 dependent. We therefore introduced Nrf2 knockdown by small interference RNAs (siNrf2) to evaluate the effectiveness of CT in regulating the ER induced apoptosis upon UVA exposure.

As shown in Figure 5A, treatment with 50 and 100 μg/mL of CT resulted in a significant decrease in pro-apoptotic signaling markers p-JNK and GADD34, as well as apoptosis-related markers, including cleaved caspase-3, -9, and the BAX/BCL2 ratio. Additionally, nuclear NRF2 and its downstream target HO-1 were elevated upon CT treatment in both concentrations. Notably, siNrf2 eliminated these effects in 100 μg/mL of CT treated with UVA, leading to increase expression of ER stress markers (ATF6, CHOP and p-eIF2α), pro-apoptotic signaling markers and apoptotic-related markers. Furthermore, Nrf2 knockdown attenuated the anti-apoptotic effects of CT (100 μg/mL), as demonstrated in Figure 5B.

Additionally, we retrieved our bulk mRNA sequencing data and focused on the expression of Nrf2 downstream antioxidant and detoxification target genes (Song et al., 2021). The heatmap generated by SRplot (Figure 5C) revealed that most genes related to antioxidation and detoxification were significantly elevated following CT treatment upon UVA exposure.

Collectively, these results imply a central role of Nrf2 in modulating ER stress induced apoptosis during CT functions.

### 3.6 Therapeutic effects of CT on macroscopic and histological changes induced by ER stress in UVA-photodamaged mouse skin

**FIGURE 6:**
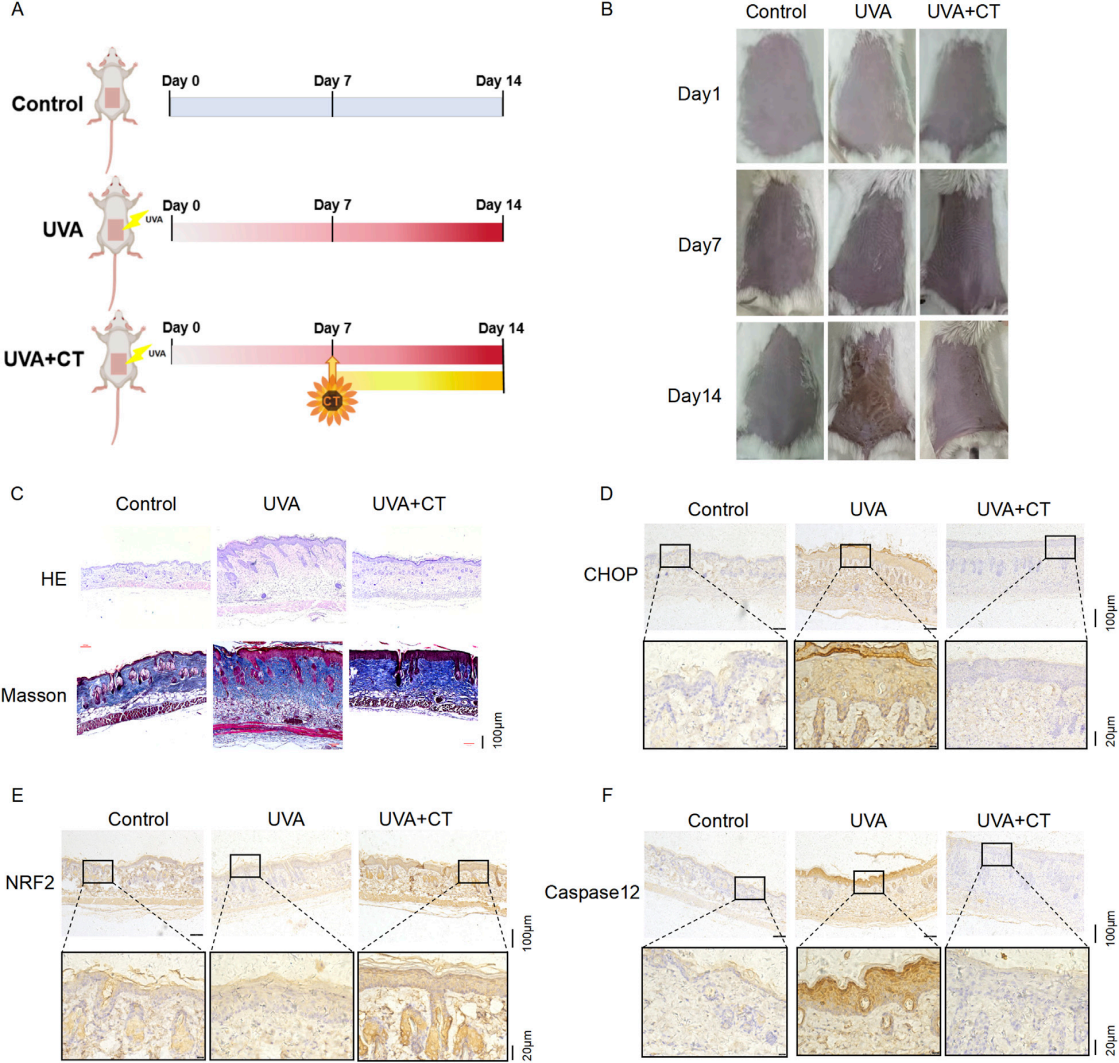
Therapeutic effects of CT on macroscopic and histological changes induced by ER stress in UVA-photodamaged mouse skin. **(A)** Schematic representation of the experimental design for mice study. **(B)** Representative pictures of untreated or treated with UVA with CT or not. **(C)** Representative pictures of H&E and Masson staining of the skin of mice from different treatment groups on day 14. **(D–F)** Representative images of CHOP, NRF2, and caspase-12 for IHC analysis performed on skin sections of mice from different treatment groups on day 14.

Our previous findings indicated that CT could reduce UVA-induced photodamage via ER stress in HaCaT cells. Therefore, we established a UVA irradiation mouse model to evaluate the effect of CT *in vivo* (Figure 6A). While mice in the control group did not receive UVA, those in the UVA or CT groups were treated with 5 J/cm^2^ UVA starting on day 0. After 14 days of UVA exposure, macroscopic photos of the dorsal skin in UVA-exposed mice showed wrinkles, redness, and desquamation. In contrast, mice treated with cream containing CT demonstrated recovery from these UVA-induced skin changes (Figure 6B).

Histological analysis was performed using H&E staining and Masson staining (Figure 6C). These results indicated that on day 14, CT treatment inhibited UVA-induced hyperplastic epidermis with hyperkeratosis and promoted well-organized collagen fibers, suggesting that CT effectively prevents UVA-induced skin photodamage.

We also assessed the protein expression levels of CHOP, caspase-12, and NRF2 using IHC staining (Figures 6D–F). The results showed that CT treatment significantly decreased the expression of CHOP and caspase-12 while promoting NRF2 expression compared to UVA-exposed mice. These findings were consistent with our previous *in vitro* results, suggesting that CT exhibits skin-protective effects against UVA irradiation by mitigating ER stress-induced apoptosis.

## 4 Discussion

While UVA rays posses less energy than UVB rays, their greater prevalence and deeper skin penetration enable significant damage to both epidermal and dermal structures, contributing to the signs of photodamage and photoaging. While UVB directly affects DNA, UVA primarily causes harm through indirect mechanisms through generating reactive oxygen species (ROS) via the activation of natural photosensitizers in the skin, which lead to genotoxic effects (Battie and Verschoore, 2012) and keratinocyte apoptosis (Tang et al., 2021). This oxidative cascade sugested the supplementation of antioxidants that counteract UVA-induced skin injury. Botanical extracts rich in phytoconstituents have emerged as promising candidates to combat UVA skin photodamage (Skarupova et al., 2020). For example, silymarin from *Silybum marianum* (L.) has been demonstrated to reduce glutathione (GSH) depletion and ROS production in UVA-treated HaCaT cells (Svobodova et al., 2007). Additionally, the flavonoid taxifolin has also been confirmed to exhibit protective activity against UVA photodamage by stimulating Nrf2 nuclear translocation and the expression of antioxidant proteins in both human skin keratinocytes and fibroblasts (Rajnochova Svobodova et al., 2022). Recently, a study on the extract of *Coreopsis tinctoria* has emerged, demonstrating that its bioactive metabolite okanin could alleviate UVB-induced photodamage in hairless mice (Sun et al., 2024). However, there is currently no study revealing the effect of CT in alleviating UVA irradiation and delineating the underlying cellular mechanisms. Here, we provide a comprehensive study to elucidate the protective effects of CT extract in mitigating UVA-induced photodamage in HaCaT cells.

With the aid of bulk RNA sequencing of UVA-irradiated HaCaT cells treated with CT reveal a unique anti-photodamage gene signature, featuring coordinated suppression of ER stress responses. While the ER stress-activated unfolded protein response (UPR) serve as a protective mechanism against the accumulation of unfolded/misfolded proteins accumulation, ustained or severe ER stress could triggers cytotoxic signaling and leading to apoptosis (Gorman et al., 2012). UV exposure induces ER stress through the generation of ROS, leading to the activation of the UPR and subsequent cell damage, including apoptosis cascades and loss of mitochondrial membrane potential (Farrukh et al., 2014; Yao et al., 2013). Indeed, Komori et al. have revealed that UVA could activate these pathways in human dermal fibroblasts simultaneously by activating transcription factor pATF6α(N), inducing IRE1-mediated splicing of XBP1 mRNA, and PERK-mediated phosphorylation of eIF2, suggesting its activation as a synergistic role in stress adaptation (Komori et al., 2012).

ER stress elicitation pathway can be divided into three predominant signaling transduction mechanisms: IRE1α, PERK, and ATF6 (Zeeshan et al., 2016). Under prolonged ER stress, both three key sensors initiate pro-apoptotic signaling cascades through the activation of the downstream molecule CHOP. While CHOP remains minimally expressed during normal homeostasis, its levels accumulates when the cell’s protective mechanisms are overwhelmed during unresolved ER stress, leading to ER stress-induced apoptosis (Li et al., 2014). Genotoxic stresses, such as UV irradiation and alkylating agents have been shown to induce CHOP expression (Ron and Habener, 1992). The PERK-eIF2α pathway is one of the primary mechanisms that regulate CHOP protein expression. This process begins with the activation of PERK, which increases the phosphorylation of eIF2α, resulting in a global reduction in protein synthesis and subsequently triggers CHOP expression (Li et al., 2014). While ATF6 has traditionally been viewed as a pro-survival signal against ER stress, it can also induce apoptosis indirectly by downregulating the expression of the anti-apoptotic protein MCL-1 (Morishima et al., 2011). Meanwhile, IRE1 exhibits both pro- and anti-apoptotic functions as part of the UPRosome complex, where its kinase and RNase activities are modulated in parallel (Gorman et al., 2012). During prolonged ER stress, IRE1’s RNase domain facilitates mRNA decay (RIDD), targeting mRNAs that encode essential ER-resident and secreted proteins, thereby promoting apoptosis (Hollien et al., 2009). IRE1 also activates JNK, leading to the stabilization of BIM, the displacement of anti-apoptotic BCL-2 proteins, and BAX/BAK-mediated apoptosis (Gorman et al., 2012). Downstream execution involves caspase-12, -9, and -3 activation, though mitochondrial permeability transition and cytochrome c release may augment this process during severe stress (Momoi, 2004; Gupta et al., 2010). Integrating these ER-related pathways offers a comprehensive understanding of how apoptosis is managed at the cellular level. In our study, we found that CT significantly influence the protein expression of phosphorylated IRE1α, phosphorylated eIF2α, and ATF6, which subsequently affect CHOP expression. Additionally, CT modulated the expression of phosphorylated JNK, BCL-2 family proteins and caspases, highlighting their potential to alleviate UVA-induced photodamage by regulating the ER stress-induced apoptosis pathway.

Accumulating evidence demonstrates the anti-apoptotic potential of plant-derived metabolites associated with ER stress. For instance, leaf extract from *Albizia lebbeck* exhibited neuroprotective effects by mitigating glutamate-induced ER stress and apoptosis in human microglial HMC3 cells (Phoraksa et al., 2023). In the context of UV-induced photodamage, *Panax ginseng* extract has been found to protect against UVB-induced skin damage by modulating VMP1-mediated ER stress (Chen et al., 2024). Other studies have identified natural metabolites, such as salubrinal and rosmarinic acid, that can alleviate UV-induced ER stress, restore calcium homeostasis, and enhance cell survival, indicating a protective role of controlled ER stress responses (Ji et al., 2017; Gupta et al., 2023). Collectively, these preclinical findings, including our demonstration of CT’s ER stress-apoptosis modulation, underscore the imperative for clinical translation of ER stress- and apoptosis-targeted therapies.

The transcription factor Nrf2 maintains cellular homeostasis by activating antioxidant proteins including HO-1, through specific binding to antioxidant response elements (AREs) (Shah et al., 2010). Previous research has indicated that the Nrf2/HO-1 axis can interact with the PERK pathway to modulate ER stress in the hypoxia-reoxygenation model (Wang et al., 2020). For instance, the flavonoid naringenin has been shown to protect kidneys from ischemia-reperfusion injury by reducing pyroptosis and apoptosis through the suppression of ER stress, achieved via activation of the Nrf2/HO-1 signaling pathway (Zhang et al., 2022). Furthermore, relevant studies have also shown that, in addition to regulating oxidative stress, the Nrf2 plays an important role in regulating apoptosis and inflammation (Sun et al., 2021; Wang et al., 2017). Our study further demonstrates that CT enhances protection against UVA-induced ER stress and photodamage through Nrf2 activation.

In summary, this work identifies CT extract as a protective agent against UVA photodamage that concurrently targets ER stress and activates Nrf2, providing a mechanistic basis for its further development.

## 5 Conclusion

**FIGURE 7:**
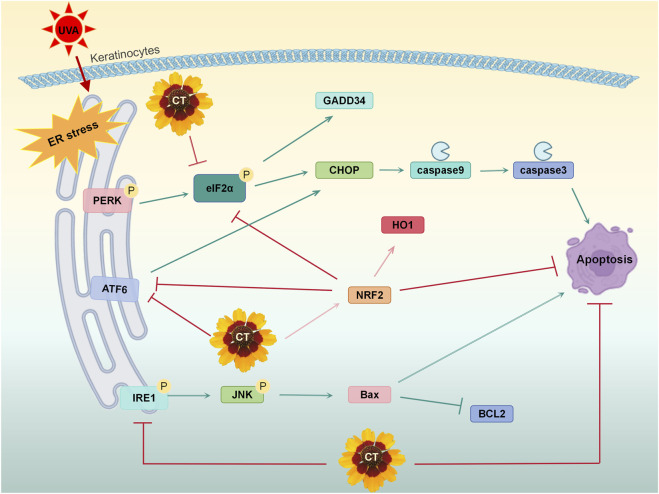
Schematic diagram illustrating the mechanism of CT in mitigating UVA-induced apoptosis by ER stress.

In conclusion, our research demonstrates that CT, a flavonoid-rich natural plant extract, is a potent mitigator of UVA-induced photodamage. It suppresses ROS, inhibits cellular apoptosis, and restores mitochondrial membrane potential both in human keratinocytes. Notably, CT effectively alleviates ER-associated apoptosis caused by UVA by broadly inhibiting all three major branches of the UPR pathway (Figure 7). We establish that Nrf2 activation plays a critical role in CT’s effects, primarily through its partial mediation of ER stress suppression. Complementary *in vivo* studies showed that topical CT treatment significantly reduced photodamage in dorsal skin. These findings support the potential of CT for clinical applications in dermocosmetics and pharmaceuticals for UV-induced skin injury. However, the phytochemical characterization remains incomplete, as quantitative composition data are lacking. To strengthen the scientific rigor and impact of this work, our future studies should focus on comprehensive qualitative and quantitative chemical characterization of the extract, alongside deeper investigation into the relationship between its chemical constituents and observed biological activities. Such efforts would provide a more solid foundation for further development and application of CT extract in skin repair and related fields.

## Data Availability

The bulk mRNA sequencing data presented in this study can be found in the Gene Expression Omnibus (GEO), accession number GSE296781. Further inquiries can be directed to the corresponding authors.

## References

[B1] Ansary T. M. Hossain M. R. Kamiya K. Komine M. Ohtsuki M. (2021). Inflammatory molecules associated with ultraviolet radiation-mediated skin aging. Int. J. Mol. Sci. 22 (8), 3974. 10.3390/ijms22083974 33921444 PMC8069861

[B2] Battie C. Verschoore M. (2012). Cutaneous solar ultraviolet exposure and clinical aspects of photodamage. Indian J. Dermatol Venereol. Leprol. 78 (Suppl. 1), S9–S14. 10.4103/0378-6323.97350 22710112

[B3] Battie C. Jitsukawa S. Bernerd F. Del Bino S. Marionnet C. Verschoore M. (2014). New insights in photoaging, UVA induced damage and skin types. Exp. Dermatol 23 (Suppl. 1), 7–12. 10.1111/exd.12388 25234829

[B4] Cadet J. Douki T. Ravanat J. L. Di Mascio P. (2009). Sensitized formation of oxidatively generated damage to cellular DNA by UVA radiation. Photochem Photobiol. Sci. 8 (7), 903–911. 10.1039/b905343n 19582264

[B5] Chen M. Li J. Xiao W. Sun L. Tang H. Wang L. (2006). Protective effect of resveratrol against oxidative damage of UVA irradiated HaCaT cells. Zhong nan da xue xue bao Yi xue ban= J. Central South Univ. Med. Sci. 31 (5), 635–639. 17062920

[B6] Chen J. Tai M. Chen J. Ni J. Yi H. Chen L. (2024). Panax ginseng extract prevents UVB-induced skin photodamage by modulating VMP1-mediated ER stress. Phytomedicine 134, 156010. 10.1016/j.phymed.2024.156010 39232284

[B7] D’Orazio J. Jarrett S. Amaro-Ortiz A. Scott T. (2013). UV radiation and the skin. Int. Journal Molecular Sciences 14 (6), 12222–12248. 10.3390/ijms140612222 23749111 PMC3709783

[B8] Farrukh M. R. Nissar U. A. Afnan Q. Rafiq R. A. Sharma L. Amin S. (2014). Oxidative stress mediated Ca(2+) release manifests endoplasmic reticulum stress leading to unfolded protein response in UV-B irradiated human skin cells. J. Dermatol Sci. 75 (1), 24–35. 10.1016/j.jdermsci.2014.03.005 24794973

[B9] Gorman A. M. Healy S. J. Jäger R. Samali A. (2012). Stress management at the ER: regulators of ER stress-induced apoptosis. Pharmacol. & Therapeutics 134 (3), 306–316. 10.1016/j.pharmthera.2012.02.003 22387231

[B10] Greinert R. Volkmer B. Henning S. Breitbart E. W. Greulich K. O. Cardoso M. C. (2012). UVA-induced DNA double-strand breaks result from the repair of clustered oxidative DNA damages. Nucleic Acids Res. 40 (20), 10263–10273. 10.1093/nar/gks824 22941639 PMC3488256

[B11] Guo L. Zhang W. Li S. Ho C.-T. (2015). Chemical and nutraceutical properties of Coreopsis tinctoria. J. Functional Foods 13, 11–20. 10.1016/j.jff.2014.11.011

[B12] Guo Y. Shen D. Zhou Y. Yang Y. Liang J. Zhou Y. (2021). Deep learning-based morphological classification of endoplasmic reticulum under stress. Front. Cell Dev. Biol. 9, 767866. 10.3389/fcell.2021.767866 35223863 PMC8865080

[B13] Gupta S. Cuffe L. Szegezdi E. Logue S. E. Neary C. Healy S. (2010). Mechanisms of ER stress-mediated mitochondrial membrane permeabilization. Int. J. Cell Biol. 2010, 170215. 10.1155/2010/170215 20169117 PMC2821636

[B14] Gupta D. Sharma R. R. Rashid H. Bhat A. M. Tanveer M. A. Abdullah S. T. (2023). Rosmarinic acid alleviates ultraviolet-mediated skin aging via attenuation of mitochondrial and ER stress responses. Exp. Dermatol 32 (6), 799–807. 10.1111/exd.14773 36811401

[B15] Hetz C. Zhang K. Kaufman R. J. (2020). Mechanisms, regulation and functions of the unfolded protein response. Nat. Rev. Mol. Cell Biol. 21 (8), 421–438. 10.1038/s41580-020-0250-z 32457508 PMC8867924

[B16] Hollien J. Lin J. H. Li H. Stevens N. Walter P. Weissman J. S. (2009). Regulated Ire1-dependent decay of messenger RNAs in mammalian cells. J. Cell Biol. 186 (3), 323–331. 10.1083/jcb.200903014 19651891 PMC2728407

[B17] Jaszewska E. Soin M. Filipek A. Naruszewicz M. (2013). UVA-induced ROS generation inhibition by Oenothera paradoxa defatted seeds extract and subsequent cell death in human dermal fibroblasts. J. Photochem. Photobiol. B Biol. 126, 42–46. 10.1016/j.jphotobiol.2013.07.001 23892189

[B18] Ji C. Yang B. Huang S. Y. Huang J. W. Cheng B. (2017). Salubrinal protects human skin fibroblasts against UVB-induced cell death by blocking endoplasmic reticulum (ER) stress and regulating calcium homeostasis. Biochem. Biophys. Res. Commun. 493 (4), 1371–1376. 10.1016/j.bbrc.2017.10.012 28988108

[B19] Jiang H. Li Z. Jiang X. Qin Y. (2022). Comparison of metabolome and transcriptome of flavonoid biosynthesis in two colors of Coreopsis tinctoria nutt. Front. Plant Sci. 13, 810422. 10.3389/fpls.2022.810422 35356116 PMC8959828

[B20] Komori R. Taniguchi M. Ichikawa Y. Uemura A. Oku M. Wakabayashi S. (2012). Ultraviolet a induces endoplasmic reticulum stress response in human dermal fibroblasts. Cell Struct. Funct. 37 (1), 49–53. 10.1247/csf.11041 22251794

[B21] Li Y. Guo Y. Tang J. Jiang J. Chen Z. (2014). New insights into the roles of CHOP-induced apoptosis in ER stress. Acta Biochim. Biophys. Sin. 46 (8), 629–640. 10.1093/abbs/gmu048 25016584

[B22] Li J. Liu D. Wu J. Zhang D. Cheng B. Zhang Y. (2016). Ginsenoside Rg1 attenuates ultraviolet B-induced glucocortisides resistance in keratinocytes via Nrf2/HDAC2 signalling. Sci. Rep. 6 (1), 39336. 10.1038/srep39336 27982079 PMC5159887

[B23] Li Q. Wang D. Bai D. Cai C. Li J. Yan C. (2020). Photoprotective effect of Astragalus membranaceus polysaccharide on UVA-induced damage in HaCaT cells. PloS One 15 (7), e0235515. 10.1371/journal.pone.0235515 32692781 PMC7373302

[B24] Li Y. Zhang J. Yan C. Chen Q. Xiang C. Zhang Q. (2022). Marein prevented LPS-Induced osteoclastogenesis by regulating the NF-kappaB pathway *in vitro* . J. Microbiol. Biotechnol. 32 (2), 141–148. 10.4014/jmb.2109.09033 35001005 PMC9628836

[B25] Ma Y. Hendershot L. M. (2004). ER chaperone functions during normal and stress conditions. J. Chem. Neuroanat. 28 (1-2), 51–65. 10.1016/j.jchemneu.2003.08.007 15363491

[B26] Ma P. Zhang R. Xu L. Liu H. Xiao P. (2022). The neuroprotective effects of coreopsis tinctoria and its mechanism: interpretation of network pharmacological and experimental data. Front. Pharmacol. 12, 791288. 10.3389/fphar.2021.791288 35222009 PMC8874282

[B27] Momoi T. (2004). Caspases involved in ER stress-mediated cell death. J. Chem. Neuroanat. 28 (1-2), 101–105. 10.1016/j.jchemneu.2004.05.008 15363495

[B28] Montes de Oca M. K. Pearlman R. L. McClees S. F. Strickland R. Afaq F. (2017). Phytochemicals for the prevention of photocarcinogenesis. Photochem Photobiol. 93 (4), 956–974. 10.1111/php.12711 28063168 PMC5500428

[B29] Morishima N. Nakanishi K. Nakano A. (2011). Activating transcription factor-6 (ATF6) mediates apoptosis with reduction of myeloid cell leukemia sequence 1 (Mcl-1) protein *via* induction of WW domain binding protein 1. J. Biol. Chem. 286 (40), 35227–35235. 10.1074/jbc.M111.233502 21841196 PMC3186435

[B30] Petruk G. Del Giudice R. Rigano M. M. Monti D. M. (2018). Antioxidants from plants protect against skin photoaging. Oxidative Medicine Cellular Longevity 2018 (1), 1454936. 10.1155/2018/1454936 30174780 PMC6098906

[B31] Phoraksa O. Chimkerd C. Thiyajai P. Judprasong K. Tuntipopipat S. Tencomnao T. (2023). Neuroprotective effects of Albizia lebbeck (L.) benth. Leaf extract against glutamate-induced endoplasmic reticulum stress and apoptosis in human microglial cells. Pharm. (Basel) 16 (7), 989. 10.3390/ph16070989 37513900 PMC10384906

[B32] Rajnochova Svobodova A. Rysava A. Cizkova K. Roubalova L. Ulrichova J. Vrba J. (2022). Effect of the flavonoids quercetin and taxifolin on UVA-induced damage to human primary skin keratinocytes and fibroblasts. Photochem Photobiol. Sci. 21 (1), 59–75. 10.1007/s43630-021-00140-9 34837635

[B33] Re R. Pellegrini N. Proteggente A. Pannala A. Yang M. Rice-Evans C. (1999). Antioxidant activity applying an improved ABTS radical cation decolorization assay. Free Radical Biology Medicine 26 (9-10), 1231–1237. 10.1016/s0891-5849(98)00315-3 10381194

[B34] Ron D. Habener J. F. (1992). CHOP, a novel developmentally regulated nuclear protein that dimerizes with transcription factors C/EBP and LAP and functions as a dominant-negative inhibitor of gene transcription. Genes & Development 6 (3), 439–453. 10.1101/gad.6.3.439 1547942

[B35] Shah Z. A. Li R.-c. Ahmad A. S. Kensler T. W. Yamamoto M. Biswal S. (2010). The flavanol (−)-epicatechin prevents stroke damage through the Nrf2/HO1 pathway. J. Cereb. Blood Flow & Metabolism 30 (12), 1951–1961. 10.1038/jcbfm.2010.53 20442725 PMC3002885

[B36] Shen J. Hu M. Tan W. Ding J. Jiang B. Xu L. (2021). Traditional uses, phytochemistry, pharmacology, and toxicology of Coreopsis tinctoria Nutt.: a review. J. Ethnopharmacol. 269, 113690. 10.1016/j.jep.2020.113690 33309917

[B37] Skarupova D. Vostalova J. Rajnochova Svobodova A. (2020). Ultraviolet A protective potential of plant extracts and phytochemicals. Biomed. Pap. Med. Fac. Univ. Palacky. Olomouc Czech Repub. 164 (1), 1–22. 10.5507/bp.2020.010 32188958

[B38] Song M. Y. Lee D. Y. Chun K. S. Kim E. H. (2021). The role of NRF2/KEAP1 signaling pathway in cancer metabolism. Int. J. Mol. Sci. 22 (9), 4376. 10.3390/ijms22094376 33922165 PMC8122702

[B39] Sun W. Wang Z. Sun M. Huang W. Wang Y. Wang Y. (2021). Aloin antagonizes stimulated ischemia/reperfusion-induced damage and inflammatory response in cardiomyocytes by activating the Nrf2/HO-1 defense pathway. Cell Tissue Res. 384 (3), 735–744. 10.1007/s00441-020-03345-z 33502605

[B40] Sun S. Li M. Wang M. Zheng J. Yin C. Wu Z. (2024). Anti-photoaging effect and the mechanism of Coreopsis tinctoria okanin against UVB-induced skin damage in mice. Int. Immunopharmacol. 139, 112657. 10.1016/j.intimp.2024.112657 39024749

[B41] Svobodova A. Zdarilova A. Maliskova J. Mikulkova H. Walterova D. Vostalova J. (2007). Attenuation of UVA-induced damage to human keratinocytes by silymarin. J. Dermatol Sci. 46 (1), 21–30. 10.1016/j.jdermsci.2006.12.009 17289350

[B42] Tai M. Chen J. Chen J. Shen X. Ni J. (2024). Endoplasmic reticulum stress in skin aging induced by UVB. Exp. Dermatol 33 (1), e14956. 10.1111/exd.14956 37846942

[B43] Tang Z. Tong X. Huang J. Liu L. Wang D. Yang S. (2021). Research progress of keratinocyte‐programmed cell death in UV‐induced skin photodamage. Photodermatol. Photoimmunol. & Photomed. 37 (5), 442–448. 10.1111/phpp.12679 33738849

[B44] Tang D. Chen M. Huang X. Zhang G. Zeng L. Zhang G. (2023). SRplot: a free online platform for data visualization and graphing. PLoS One 18 (11), e0294236. 10.1371/journal.pone.0294236 37943830 PMC10635526

[B45] Wang L. Yao Y. He R. Meng Y. Li N. Zhang D. (2017). Methane ameliorates spinal cord ischemia-reperfusion injury in rats: Antioxidant, anti-inflammatory and anti-apoptotic activity mediated by Nrf2 activation. Free Radic. Biol. Med. 103, 69–86. 10.1016/j.freeradbiomed.2016.12.014 28007572

[B46] Wang J. Lu L. Chen S. Xie J. Lu S. Zhou Y. (2020). PERK overexpression-mediated Nrf2/HO-1 pathway alleviates Hypoxia/Reoxygenation-Induced injury in neonatal murine cardiomyocytes via improving endoplasmic reticulum stress. Biomed. Res. Int. 2020, 6458060. 10.1155/2020/6458060 32309436 PMC7136769

[B47] Wang Y. Cheng J. Jiang W. Chen S. (2022). Metabolomics study of flavonoids in Coreopsis tinctoria of different origins by UPLC–MS/MS. PeerJ 10, e14580. 10.7717/peerj.14580 36570002 PMC9774014

[B48] Yao J. Bi H. E. Sheng Y. Cheng L. B. Wendu R. L. Wang C. H. (2013). Ultraviolet (UV) and hydrogen peroxide activate ceramide-ER stress-AMPK signaling axis to promote retinal pigment epithelium (RPE) cell apoptosis. Int. J. Mol. Sci. 14 (5), 10355–10368. 10.3390/ijms140510355 23685869 PMC3676843

[B49] Yu H. Jiang G. Hu W. Xu C. (2022). Pin1 aggravates renal injury induced by ischemia and reperfusion in rats via Nrf2/HO-1 mediated endoplasmic reticulum stress. Acta Cir. Bras. 37 (1), e370101. 10.1590/acb370101 35416857 PMC9000979

[B50] Zagórska-Dziok M. Mokrzyńska A. Ziemlewska A. Nizioł-Łukaszewska Z. Sowa I. Feldo M. (2024). Assessment of the antioxidant and photoprotective properties of cornus mas L. extracts on HDF, HaCaT and A375 cells exposed to UVA radiation. Int. J. Mol. Sci. 25 (20), 10993. 10.3390/ijms252010993 39456776 PMC11507244

[B51] Zeeshan H. M. Lee G. H. Kim H. R. Chae H. J. (2016). Endoplasmic Reticulum stress and associated ROS. Int. J. Mol. Sci. 17 (3), 327. 10.3390/ijms17030327 26950115 PMC4813189

[B52] Zhang B. Wan S. Liu H. Qiu Q. Chen H. Chen Z. (2022). Naringenin alleviates renal ischemia reperfusion injury by suppressing er stress‐induced pyroptosis and apoptosis through activating nrf2/ho‐1 signaling pathway. Oxidative Med. Cell. Longev. 2022 (1), 5992436. 10.1155/2022/5992436 36262286 PMC9576412

[B53] Zhong J. Liang L. Zhao N. Wang J. Shu P. (2025). Synergistic effects of retinol and retinyl palmitate in alleviating UVB-induced DNA damage and promoting the homologous recombination repair in keratinocytes. Front. Pharmacol. 16, 1562244. 10.3389/fphar.2025.1562244 40343007 PMC12058701

[B54] Zhu X. Li N. Wang Y. Ding L. Chen H. Yu Y. (2017). Protective effects of quercetin on UVB irradiation-induced cytotoxicity through ROS clearance in keratinocyte cells. Oncol. Rep. 37 (1), 209–218. 10.3892/or.2016.5217 27840962

