# Supplementary material for: Coreopsis tinctoria Nutt. attenuates ultraviolet A photodamage by suppressing endoplasmic reticulum stress-induced apoptosis via Nrf2 crosstalk
Source: Front Pharmacol. 2025 Dec 12;16:1686234. doi: 10.3389/fphar.2025.1686234 (PMC12741067; doi:10.3389/fphar.2025.1686234)
Supplement: Supplementary file 1 [file Table1.docx]

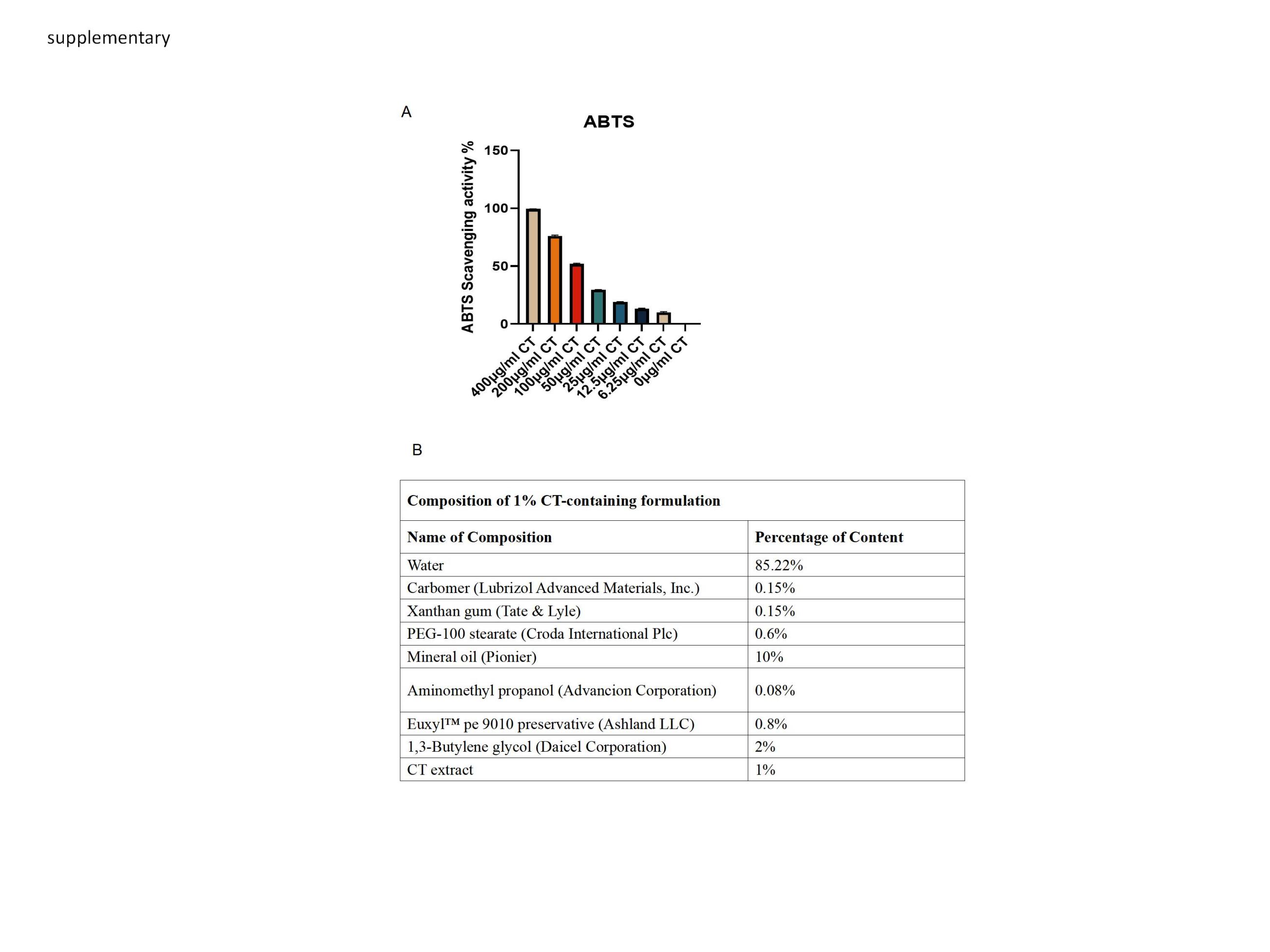


**Supplementary Figure 1**

(A) ABTS assay was performed to determine the antioxidant capacity of CT extract. N=4.

(B) Composition of 1% CT-containing formulation used in the mouse experiments.
